# An Artificial miRNA against HPSE Suppresses Melanoma Invasion Properties, Correlating with a Down-Regulation of Chemokines and MAPK Phosphorylation

**DOI:** 10.1371/journal.pone.0038659

**Published:** 2012-06-15

**Authors:** Xiaoyan Liu, Hong Fang, Hongchao Chen, Xiaoling Jiang, Deren Fang, Yan Wang, Dingxian Zhu

**Affiliations:** Department of Dermatology, The First Affiliated Hospital, College of Medicine, Zhejiang University, Hangzhou, People's Republic of China; University of Chicago, United States of America

## Abstract

Ribonucleic acid interference (RNAi) based on microRNA (miRNA) context may provide an efficient and safe therapeutic knockdown effect and can be driven by ribonucleic acid polymerase II (RNAP II). In this study, we designed and synthesized miR155-based artificial miRNAs against heparanase (HPSE) constructed with BLOCK-iT™ Pol II miR RNAi Expression Vector Kit. The expression levels of HPSE declined significantly in both the mRNA and protein levels in HPSE-miRNA transfected melanoma cells that exhibited reduction of adhesion, migration, and invasion ability *in vitro* and *in vivo*. We also observed that HPSE miRNA could inhibit the expressions of chemokines of interleukin-8 (*IL8*) and chemokine (C-X-C motif) ligand 1 (*CXCL1)*, at both the transcriptional and translational levels. Further study on its probable mechanism declared that down-regulation of *IL8* and *CXCL1* by HPSE-miRNA may be correlated with reduced growth-factor simulated mitogen-activated kinase (MAPK) phosphorylation including p38 MAPK, c-Jun N-terminal kinase (JNK) and extracellular-signal-regulated kinase (ERK) 1 and 2, which could be rescued by miRNA incompatible mutated HPSE cDNA. In conclusion, we demonstrated that artificial miRNAs against *HPSE* might serve as an alterative mean of therapy to low HPSE expression and to block the adhesion, invasion, and metastasis of melanoma cells. Furthermore, miRNA-based RNAi was also a powerful tool for gene function study.

## Introduction

Malignant melanoma is one of the most aggressive and fastest increasing cancers with a high mortality and a poor prognosis [1]. Metastatic melanoma is difficult to treat with current therapies. Therefore, it is of great significance to improve our understanding of the complex molecular mechanisms of its invasive and metastatic potential, and to further develop therapeutic modalities for ideal targets to improve the survival rates of melanoma patients.

HPSE is an endo-β-glucuronidase that can cleave heparan sulfate proteoglycans (HPSG) within the extracellular matrix (ECM), basement membrane (BM) or on the cellular surface, facilitating metastasis by enhancing cell invasion, migration, intravasation and extravasation [2]. Elevated expression of HPSE in tumor cells dramatically enhances their growth, angiogenesis, and metastasis to bone or brain [3]–[4]. PI-88, a potent HPSE inhibitor, has shown antitumor activity *in vitro* and *in vivo* by inhibiting vascular endothelial growth factor and fibroblast growth factor, directly or indirectly, as well as stimulating the release of tissue factor pathway inhibitors [5]. Promising results from Phase I/II trials are being seen with PI-88 in a variety of tumor types. However, the development of antibody-induced thrombocytopenia or neutropenia has limited the use of PI-88 in some patients [5]–[6]. Therefore, there is still an unmet need for identifying and developing a novel therapy to prevent the progression of tumors without sacrificing patient quality of life.

RNAi is an attractive technology for the knockdown of specific genes and is being developed as a therapeutic modality [7]. To date, most expression-based RNAi strategies have utilized small interfering RNA (siRNA) or short hairpin RNA (shRNA). The use of siRNA (diced siRNA or synthetic siRNA) for RNAi analysis in mammalian cells is limited by their transient nature and the lack of an efficient delivery system *in vivo* [8], [9]. The use of shRNA requires the screening of a large number of sequences to identify active sequences, and the use of Pol III promoters limits applications such as tissue-specific expression [10], [11]. miRNAs, which endogenously express small ssRNA sequences of ∼22 nucleotides, can naturally direct gene silencing through components shared with the RNAi pathway [12]. Recently, it was reported that using the endogenous processing machinery, optimized shRNA constructs based on miRNAs may provide more efficient and safer therapeutic RNAi expression [13]–[15]. Furthermore, such a shRNA embedded in a miRNA scaffold can be driven by RNA Pol II [16], which makes tissue-specific RNAi possible [17], [18]. In this study, we constructed miR155-based artificial miRNAs against HPSE with the BLOCK-iT™ Pol II miR RNAi Expression Vector Kit to investigate their effect on HPSE down-modulation and other functions *in vitro* and *in vivo*, with the aim of exploring an efficient and safe approach for melanoma treatment.

## Materials and Methods

### Design and Synthesis of miR-155-based HPSE miRNA and the Construction of Vectors

**Figure 1:**
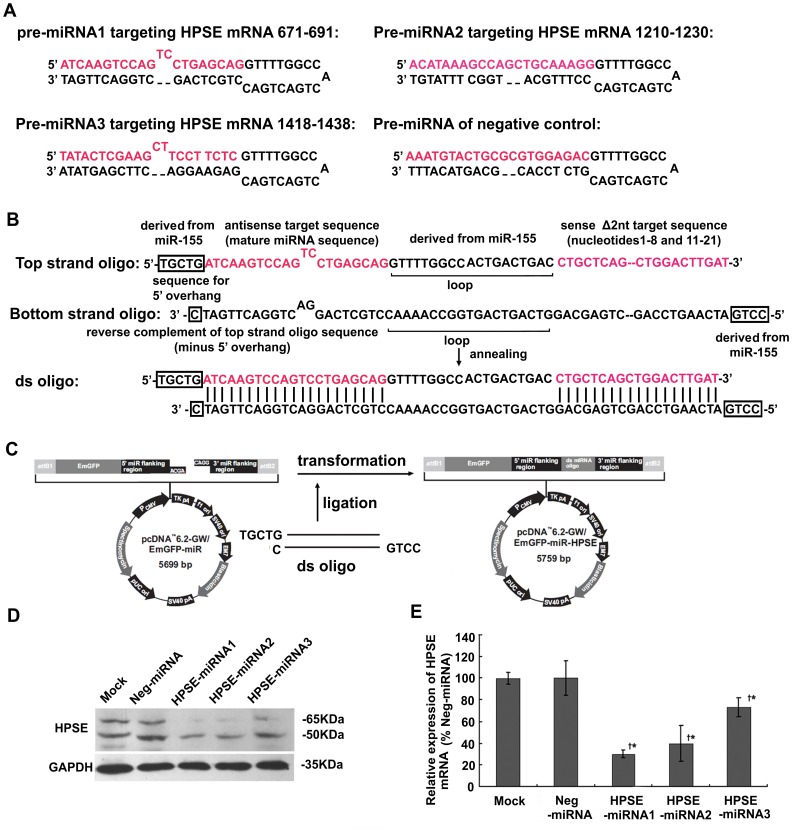
Construction of of miR-155-based HPSE miRNAs and their impact on HPSE expression levels in A375 cells. A375 cells were transfected with HPSE-miRNAs or Neg-miRNA for 48 hours. (A) The sequences and predicted secondary structures of three designed pre-miRNAs targeting *HPSE* (HPSE-miRNA1, HPSE-miRNA2, and HPSE-miRNA3) and a negative control miRNA (Neg-miRNA), and the precise regions of the *HPSE* mRNA that they targeted. (B) Pre-miRNA double-stranded oligo inserted into the miRNA expression vector-pcDNA6.2-GW/EmGFP-miR. (C) Schematic representation of the process of ligation and transformation. (D) Inhibitory effects of HPSE miRNAs on HPSE protein expression. Representative blots are shown from three independent experiments with identical results. The expressions of HPSE protein in A375 cells transfected with HPSE miRNAs were obviously down-regulated obviously compared to the parental cells and the Neg-miRNA group. (E) Inhibitory effects of HPSE miRNAs on *HPSE* mRNA expression. Calculation of the respective *HPSE* mRNA expression in each group was relative to the Neg-miRNA group (%). Quantitative real-time PCR results showed that the expression of *HPSE* mRNA in A375 cells transfected with HPSE miRNAs were down-regulated compared to the parental cells or the Neg-miRNA transfected cells. (^†^ *P*<0.05, compared with the parental cells; *P<0.05, compared with the Neg-miRNA transfected cells).

Pre-miRNA sequences for HPSE (NM_006665.3) were designed by Invitrogen’s RNAi Designer (sequences were shown in Figure 1A). The synthesized complementary DNA oligos (TaKaRa Biotechnology Co. Ltd., Dalian, China) were annealed to generate a double-stranded oligo and cloned into the linearized pcDNA™ 6.2-GW/EmGFP-miR vector (Invitrogen Corp., Carlsbad, CA, USA) using T4 DNA ligase (Figure 1B and C). The Neg-miRNA control plasmid was included in the Block-iT™-Pol II miR RNAi Expression Vector Kit (sequences were shown in Figure 1A). All of the vectors were transformed into One Shot® TOP10 Chemically Competent *E. coli* (Invitrogen Corp.), and the colonies containing spectinomycin-resistant transformants were analyzed for the desired expression clones. The recombinant vectors were purified with a purification kit (Qiagen Inc., Valencia, CA, USA) and confirmed by sequencing (TaKaRa).

### Cell Culture and Stable Transfection

The human malignant melanoma cell line A375 was purchased from the Shanghai Institute of Cell Biology (Shanghai, China) and routinely maintained in Dulbecco’s modified Eagle’s medium (DMEM) (Hyclone Laboratories, Inc., Logan, UT, USA) supplemented with 10% fetal bovine serum and 20 mmol/L of HEPES (4-(2-hydroxyethyl)-1-piperazineethanesulfonic acid) in a 5% CO_2_ incubator at 37°C. The vectors with HPSE-miRNA1, HPSE-miRNA2, HPSE-miRNA3 (targeting different sequences of HPSE as shown in Figure 1A) or the Neg-miRNA were transfected into A375 cells using Attractene Transfection Reagent (Qiagen Inc.) according to the manufacturer’s protocol. For effective screening of RNAi sequences targeting HPSE by quantitative real-time polymerase chain reaction (PCR) and western blotting, the cells were transiently transfected with each miRNA for 48 hours, when the percentage of fluorescent cells was more than 80%. For further study, the aforementioned transfectants were selected by fresh DMEM medium containing 12.5 µg/mL blasticidin (Invitrogen Corp.) every 3 to 4 days until blasticidin-resistant colonies could be identified. To confirm the effect of the HPSE-miRNA on other tumor cells with a high invasive ability, it was also transfected into cervical carcinoma HeLa cells maintained in Roswell Park Memorial Institute 1640 medium (Hyclone, Inc.).

### Quantitative Real-time PCR

Total RNA was isolated from cells of different groups of parental cells, which were transfected with Neg-miRNA, HPSE-miRNA1, HPSE-miRNA2 or HPSE-miRNA3, using RNAiso™ PLUS (TaKaRa), following the manufacturer’s protocol. The reactions were carried out in a 20 µL reaction volume containing 10 µL of 2 × SYBR® Premix Ex Taq™ and 0.4 µL of 50 × ROX reference dye (TaKaRa). Primers for *HPSE*, *IL8*, *CXCL1*, as well as the internal control *β-actin,* were synthesized by the TaKaRa Company as follows: *HPSE*-forward, 5′-GAATGGACGGACTGCTAC -3′, *HPSE*-reverse, 5′-CCAAAGAATACTTGCCTCA-3′; *IL8*-forward, 5′-ACACTG CGCCAACACAGAAATTA-3′, *IL8*-reverse, 5′-TTTGCTTGAAGTTTCACTGGCA TC-3′; *CXCL1*-forward, 5′-GAACATCCAAAGTGTGAACGTGAAG-3′; *CXCL1*-reverse, 5′-TTCAGGAACAGCCACCAGTGAG-3′; *β-actin*-forward, 5′-GG CGGCACCACCATGTACCCT-3′, *β-actin*-reverse, 5′-AGGGGCCGGACTCGTCA TACT-3′. The ΔCt data were collected automatically. −ΔΔCt was calculated by −ΔΔCt = average ΔCt of the negative control group −ΔCt of the treated group. The relative expression for a target gene was calculated using 2^−ΔΔCt^. All experiments were repeated three times.

### Western Blotting Analysis

Cells from different groups were harvested, lysed and subjected to western blotting with the antibodies for the target genes as described previously [19]. Antibodies to HPSE, anti-phospho-p38 MAPK (Thr180/Tyr182), anti-p38 MAPK, anti-phospho -JNK (Thr183/Tyr185), anti-JNK, anti-phospho-ERK1/2 (Thr202/Tyr204) and anti-ERK1/2 were obtained from Abcam® Biotechnology plc (San Francisco, CA, USA). Following 3 washes with TBS/T buffer, the membranes were incubated with horseradish peroxidase-conjugated anti-rabbit IgG for 1 hour at room temperature. EZ-ECL (Biological Industries Israel Beit-Haemek Ltd., Kibbutz Beit-Haemek, Israel) was subsequently used for visualization of the bands. The membranes were stripped and probed with the glyceraldehyde-3-phosphate dehydrogenase (GAPDH) monoclonal antibody (KangChen Bio-tech Inc., Shanghai, China), which served as the internal control. All experiments were repeated three times.

### *In vitro* Cellular Viability and Proliferation Assay

Parental cells and cells stably transfected with Neg-miRNA, HPSE-miRNA1, or HPSE-miRNA2 were seeded in 96-well plates at a density of 5×10^3^ cells per well. Each group was repeated for 5 × 3 wells. After subculturing for 1, 24, and 48 hours, 20 µL of MTT (3-(4,5-dimethylthiazol-2-yl)-2,5-diphenyltetrazolium bromide, 5 mg/mL) (Sigma-Aldrich Co. LLC, St Louis, MD, USA) was added to each well, and the plates were incubated for an additional 4 hours at 37°C. The MTT solution in the medium was then aspirated, and 150 µL of dimethyl sulfoxide (Sigma-Aldrich Co. LLC) was added before measurement of the absorbance at 570 nm. Cellular viability was evaluated by the A_570_ value. In addition, we counted the cells number of each group after 24, 48, 72, and 96 hours of culture in replicated 6-well plates, at an initial density of 1×10^5^ cells per well.

### Annexin-V-fluorescein Isothiocyanate/Propidium Iodide (Annexin-V-FITC/PI) Affinity Assay

Parental cells and cells that were stably transfected with Neg-miRNA, HPSE-miRNA1, or HPSE-miRNA2 were collected and re-suspended at 2×10^6^ cells/mL in Annexin-V binding buffer. Each experiment was performed in triplicate. The supernatant (100 µL/tube) was incubated with 5 µL of Annexin-V-FITC (Biosource, Carmarillo, CA, USA) and 5 µL of propidium iodide (Sigma-Aldrich) for 15 min at room temperature in the dark, followed by cytometric analysis (Becton, Dickinson and Company, Franklin, NJ, USA) within 1our of staining.

### Cellular Adhesion Assay

Each well of the 96-well plates was coated with Matrigel (1∶3 dilution ratio, BD) and bull serum albumin (2%, 20 µL), and dried in a Superclean Bench. Parental cells and cells stably transfected with Neg-miRNA, HPSE-miRNA1, or HPSE-miRNA2 were collected and seeded in the prepared 96-well plates at a concentration of 1 × 10^4^ per well. Each experiment was performed in quadruplicate. Cells that did not adhere to the Matrigel were washed off by phosphate-buffered saline after a 1 hour of incubation at 37°C. The cellular adhesion ability was evaluated by counting the remaining cells as detected by MTT assay.

### Transwell Migration Assay and Matrigel Invasion Assay

Cells (2×10^5^/mL) from each group (100 µL) were re-suspended in serum-free DMEM and seeded in the top chambers of non-coated chambers (24-well insert; 8 µm pore size; Corning Costar Corp., Cambridge, MA, USA). The chambers were then placed into 24-well plates, and the lower chambers were filled with 0.5 mL of DMEM medium containing 10% fetal bovine serum as a chemoattractant. After subculturing for 24 hours, the cells on the upper surface of the membrane were removed using cotton tips. The cells that migrated to the lower surface were fixed in 10% formalin at room temperature for 30 minutes and stained with hematoxylin and eosin (H&E). The cellular migration ability was determined by counting the H&E-stained cells under the light microscopy with a magnification of 100×. The ability of the cells to invade through a Matrigel-coated filter was also measured in transwell chambers, in addition that Matrigel (1∶3 dilution, BD), a reconstituted basement membrane containing HSPG, was added to the bottom of each transwell chamber.

### Gene Microarray Analysis

Total RNA were extracted from A375 cells stably transfected with Neg-miRNA, HPSE-miRNA1, and HPSE-miRNA2. The RNA samples were delivered to the LC Company (Hangzhou, China) and analyzed by the Human Whole Genome OneArray**®** v5 (Phalanx Biotech Group, Taiwan, China) for gene microarray analysis. The array contains 30,275 DNA oligonucleotide probes, 29,178 human genome probes, and 1,088 experimental control probes formed as 60-mer sense-strand DNA elements. Four hybridizations for each group were performed, with two biological and two technical replicates. Briefly, the signal intensity of each spot was loaded into the Rosetta Resolver System**®** (Rosetta Biosoftware, Cambridge, MA, USA) for data analysis. The technical repeat data were tested by Pearson’s correlation coefficient calculation to check the reproducibility (*R*>0.95). Normalized spot intensities were transformed to gene expression log_2_ ratios between the control and treatment groups. The spots with a log_2_ ratio ≥1 or log_2_ ratio ≤ −1 and *P*<0.05 were tested for further analysis. Furthermore, a gene-set enrichment analysis (GSEA, http://www.broadinstitute.org/gsea) was performed to detect pathways that are significant. All data are MIAME compliant, and the raw data have been deposited in a MIAME-compliant database (accession number E-MEXP-3443).

### ELISA Assay

Parental cells and cells stably transfected with Neg-miRNA, HPSE-miRNA1, or HPSE-miRNA2 were incubated for 48 hours, and the medium was collected and used for an enzyme-linked immunosorbent assay (ELISA). IL8 and CXCL1 ELISAs were performed according to the manufacturer’s instructions with the ELISA kit (R&D Systems® Inc., Minneapolis, MN, USA).

### *In vivo* Assays for Tumor Metastasis

All animal protocols were approved by the Animal Care and Use Committee of the Zhejiang University of Traditional Chinese Medicine (Hangzhou, China). BALB/c-nu mice (4–5 weeks old) were bred in laminar-flow cabinets and kept at a constant humidity and temperature (25–28°C). Parental cells and cells transfected stably with Neg-miRNA and HPSE-miRNA2 (200 µL, 1×10^7^/mL) were injected into the tail vain of nude mice (3 in each group). The mice were weighed once a week and sacrificed 6 weeks later, at which time, the lungs and livers were removed. Consecutive sections were made for every tissue block of the lungs or livers and stained with H&E. The incidence of lung or liver metastasis was calculated and evaluated independently by two pathologists. Furthermore, the metastases were classified into grade I-IV, according to the number of A375 cells in the metastatic lesion [20].

### Multisite-directed Mutagenesis for HPSE miRNA Rescue Experiment

We performed the multisite-directed mutagenesis test to corroborate the specificity of the phenotypic changes associated with the HPSE miRNA. The pcDNA3.1-HPSE plasmid containing the full length human cDNA was kindly provided by Dr. Israel Vlodavsky (Technion, Haifa, Israel). Primers for multisite mutagenesis were designed and synthesized by Invitrogen Corp. as follows: forward, 5′-CTATCCGACACCTTT GCAGCCGGATTCATGTGGCTGGATAAATT-3′; reverse, 5′-AATTTATCCAGCC ACATGAATCCGGCTGCAAAGGTGTCGGATAG-3′, in order; to introduce three nucleotide substitutions within the HPSE-miRNA2 hybridizing sequence (5′-CCTTTGCAGC**T**GG**C**TT**T**ATGT-3′), while retaining the amino acid identity of the wild-type protein. A detailed procedure of the mutagenesis reaction was included in the Figure S1. DNA from five colonies was isolated using a purification kit (Qiagen) and sequenced (Invitrogen Corp.) to verify the presence of the designed mutations. Original and mutant HPSE cDNAs were transfected into cells that stably expressed Neg-miRNA, HPSE-miRNA1 and HPSE-miRNA2. Total RNA, cell lysates and supernatants were harvested at 48 hours after transfection for further analysis.

### Statistical Analysis

The SAS software was used for statistical analysis. The results were expressed as the mean±standard deviation. One-way analysis of variance followed by the Dunnett-t and SNK-q tests were used to assess significant differences among the groups. *P*<0.05 was considered to be statistically significant. The results were representative of at least three independent experiments with reproducible results.

## Results

### Artificial HPSE miRNAs were Successfully Constructed, which Down-regulated HPSE Expression in Melanoma Cells

Three pcDNA6.2-GW/EmGFP-miR-based miRNA expression plasmids with the pre-miRNA sequences of human *HPSE* mRNA, termed HPSE-miRNA1, HPSE-miRNA2 and HPSE-miRNA3, were constructed (Figure 1A). The positive plasmids were cloned, sequenced, confirmed (Figure S2), and transfected into A375 cells, which expressed high levels of HPSE [3], [19]. Compared to the parental cells and the negative control (Neg-miRNA), the cells transfected with HPSE miRNAs for 48 hours demonstrated a significantly decreased expression of the HPSE protein, especially in the group of HPSE-miRNA1 and HPSE-miRNA2 groups (Figure 1D). Consistent with the western blotting results, all of the three HPSE miRNAs inhibited the expression of *HPSE* mRNA, especially the HPSE-miRNA1 and HPSE-miRNA2 (69.76% and 60.31%, respectively, *P*<0.05, Figure 1E). Thus, the A375 cells stably transfected with HPSE-miRNA1, HPSE-miRNA2 and Neg-miRNA were selected with 12.5 µg/mL blasticidin and were used for further studies. Furthermore, HPSE-miRNA1 and Neg-miRNA were also transfected into HeLa cells, which also highly expressed HPSE [21] as HPSE-miRNA1 was able to inhibit the expression of HPSE (Figure S3A and B).

### HPSE miRNAs Attenuated Cellular Viability and Proliferation, but did not Induce Apoptosis of Melanoma Cells *in vitro*

**Figure 2:**
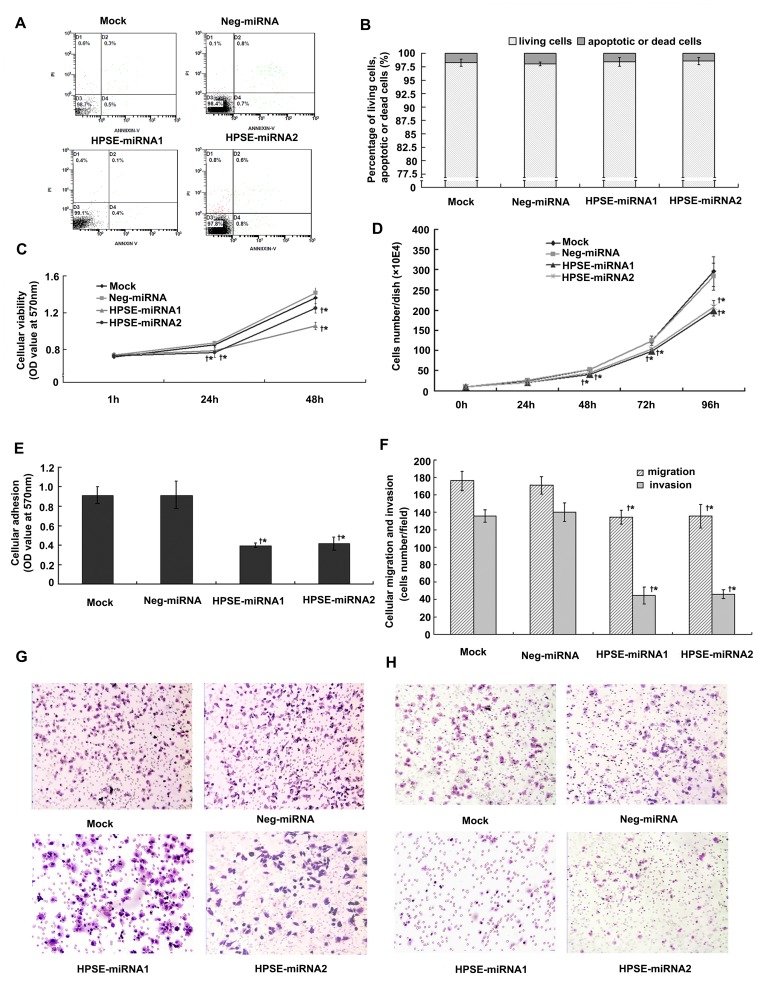
Effects of HPSE miRNAs on A375 cells *in vitro*. A375 cells transfected stably with HPSE-miRNA1, HPSE-miRNA2 or Neg-miRNA were selected by blasticidin. (A-B) Flow cytometric results showed that the percentages of apoptotic cells in both HPSE-miRNA groups were not different from those in the parental cells or the Neg-miRNA group (*P*>0.05). (C) MTT assay showed that HPSE miRNA attenuated the cellular viability of A375 cells at both 24 hours and 48 hours, compared to the parental cells or the Neg-miRNA transfected cells. (D) Cellular proliferation assay using cell counts showed that HPSE miRNA could also inhibit cellular proliferation of A375 cells at 48, 72 and 96 hours, compared to the parental cells or the Neg-miRNA transfected cells. (E) Cell-Matrigel adhesion assay. The adhesive ability of A375 cells transfected with HPSE miRNAs was obviously inhibited compared to control groups. (F) Diagram of migrative cells or invasive cells, as determined by the transwell migration assay or the Matrigel-invasion assay. The migrative or invasive number of A375 cells transfected with HPSE miRNAs was much less than that of either control group. (G) Representative images of migrative cells in the HPSE miRNAs groups or control groups in the transwell migration assay (H&E staining, magnification of 10×10). (H) Representative images of invasive cells in the HPSE miRNAs groups or control groups in Matrigel invasion assay(H&E staining, magnification of 10×10). (^†^ *P*<0.05, compared with the parental cells; *P<0.05, compared with the Neg-miRNA transfected cells).

The flow cytometry results showed that the percentages of apoptotic cells in both HPSE-miRNA groups did not differ from the parental cells or the Neg-miRNA group (*P*>0.05, Figure 2A and B). However, we found that cells transfected stably with HPSE miRNA had reduced viability when compared to either control group at 24 and 48 hours. The *A_570_* value of each group detected by MTT assay at 1 hour showed no differences (*P*>0.05). When incubated for 24 or 48 hours, the *A_570_* value of HPSE-miRNA1 and HPSE-miRNA2 was 0.787±0.011, 0.767±0.053 (24 hours) and 1.056±0.040, 1.249±0.052 (48 hours), respectively, significantly lower than both control groups (*P*<0.01) (Figure 2C). In addition, similar results were found in the cellular proliferation assay by counting cells number. Both HPSE-miRNA1 and HPSE-miRNA2 were found to inhibit cellular proliferation of A375 cells at 48, 72 and 96 hours, compared to the parental cells or Neg-miRNA cells (*P*<0.05). At the 24 hour time point, however, the cells number did not differ significantly amongst the groups (*P*>0.05).

### HPSE miRNAs Abolished the Adhesion, Migration, and Invasion of Melanoma Cells *in vitro*

**Figure 3:**
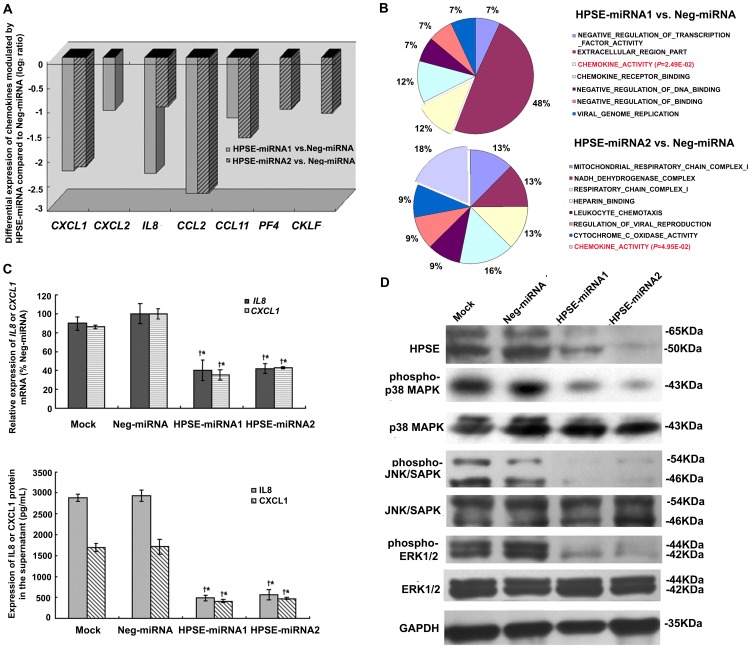
HPSE miRNAs inhibited expression of *IL8* and *CXCL1* and its probable mechanism. (A) Differentially expressed chemokine genes in the HPSE-miRNA1 and HPSE-miRNA2 groups compared to the Neg-miRNA group (log_2_ ratio≥1 or log_2_ ratio≤−1 and *P*<0.05). (B) Pathways including chemokines activity modulated by HPSE-miRNA1 or HPSE-miRNA2 were confirmed to be significant by gene-set enrichment analysis (*P*<0.05). (C) Both the mRNA and protein levels of *IL8* and *CXCL1* in HPSE miRNA transfected A375 cells were decreased compared to either control group. (^†^ *P*<0.001, compared with the parental cells; *P<0.001, compared with the Neg-miRNA transfected cells). (D) Attenuation of the HPSE-induced phosphorylation of MAPKs by HPSE miRNA. Phosphorylation of MAPK p38 (second and third panel), JNK/SAPK (fourth and fifth panel), and ERK1/2 (sixth and seventh panel) was monitored by western blotting.

Adhesion to Matrigel was evaluated by MTT assay at the indicated time point. The *A_570_* value in both control groups (0.916±0.087 and 0.916±0.142) differed significantly compared to HPSE-miRNA1 (0.398±0.022) and HPSE-miRNA2 transfected A375 cells (0.416±0.068) (*P*<0.001, Figure 2E). Furthermore, the number of A375 cells transfected with HPSE-miRNA1 or HPSE-miRNA2 that migrated to the lower surface of the transwell chambers and invaded through Matrigel at 24 hours was significantly lower than that of the A375 cells in the control groups (*P*<0.001, Figure 3F, G and H). In addition, HPSE-miRNA1 could also attenuate the adhesion, migration, and invasion ability of HeLa cells (Figure S3C, D, E and F).

### Gene Expression Changes in HPSE miRNA Transfected A375 Cells Detected by gene Microarray Assay

We analyzed and compared genes between the Neg-miRNA transfected A375 cells and HPSE-miRNA1 or HPSE-miRNA2 transfected A375 cells by gene microarray analysis using OneArray™ slides. A total of 406 (HPSE-miRNA1 transfected) and 680 (HPSE-miRNA2 transfected) genes were up or down-regulated ≥2 fold (*P*<0.05), compared with the Neg-miRNA transfected group (Figure S4A). Amongst these differentially expressed genes, 205 genes were overlapping which indicated that they are a result of HPSE knockdown, rather than consequences of off-target effects of RNAi. The 205 overlapping genes were analyzed for functional annotation by DAVID (http://david.abcc.ncifcrf.gov/) and were found to be involved in growth-factor binding, negative regulation of signal-transduction, immune response, etc., as well as wound response, extracellular region localization, heparin binding, inflammatory response, and regulation of cell migration (Figure S4B). Furthermore, chemokines (e.g., *IL8*, *CXCL1*, *CCL2* and *CCL11*) were found to be down-regulated in both HPSE miRNA groups (Figure 3A). Additionally chemokines were confirmed to be differentially expressed by gene-set enrich analysis in HPSE miRNA groups compared with the Neg-miRNA group (*P*<0.05, Figure 3B), which indicated that HPSE may play a role in the production of chemokines. Furthermore, we detected mRNA and protein expression of *IL8* or *CXCL1* by quantitative real-time PCR and ELISA assay. The data demonstrated that the mRNA and protein levels of *IL8* and *CXCL1* was decreased remarkably, compared to the parental cells and the Neg-miRNA group (*P*<0.01, Figure 3C).

### HPSE miRNAs Inhibited the Expression of *IL8* and *CXCL1*, in Part, by Attenuation of MAPK Phosphorylation

To verify whether the *IL8*/*CXCL1*/MAPK pathway was blocked due to the lack of HPSE expression with artificial HPSE miRNA, we performed western blotting for the phosphorylation of MAPK, including p38 MAPK, JNK and ERK1/2. The levels of p38 MAPK, JNK and ERK phosphorylation were reduced in HPSE-miRNA1 and HPSE-miRNA2 transfected cells as compared to Neg-miRNA transefected cells and the parental cells (Figure 3D). These results suggested an involvement of HPSE-mediated signaling on the expression of chemokines in melanoma cells.

### HPSE miRNA Suppressed Lung Metastasis of A375 Cells

**Figure 4:**
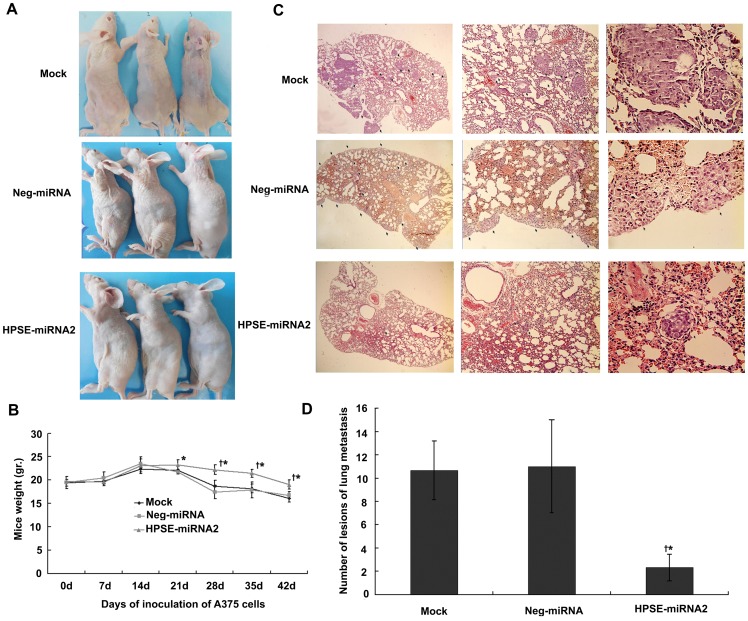
The effect of HPSE miRNA on the *in vivo* lung metastasis of A375 cells. Cells (2×10^6^) from the parental cells, Neg-miRNA or HPSE-miRNA2 transfected cells were injected into the tail veins of nude mice. (A-B) Xenograft mice were weight once a week for 6 weeks, and photos were taken at the end of the experiment on day 42, as shown in A. A weight increase was noted in the HPSE-miRNA2 group, compared to the control groups. (C) Representative lung tissue sections from each group (H&E staining, magnification of ×40, ×100 and ×400, respectively). (D) The number of lung metastases in HPSE-miRNA2 mice was decreased compared to that of mock control and Neg-miRNA control groups. (^†^ *P*<0.05, compared with the parental cells; *P<0.05, compared with the Neg-miRNA transfected cells).

At the start of the *in vivo* experiments, the weights of mice were not different amongst the groups (*P*>0.05). One days 21, 28, 35 and 42 after the inoculation of tumor cells, the weights of the mice in the HPSE-miRNA2 group were higher than both control groups (*P*<0.05, Figure 4A and B). Unexpectedly, in our experiments, there was no metastasis in the liver, another vulnerable metastatic site of melanoma (data not shown). At the end of the six weeks, the number of lung metastatic lesions in the HPSE-miRNA2 group (2.333±1.155) was much less than that in the control groups (10.667±2.216 and 11.000±4.000) (*P*<0.05, Figure 4C and D). Furthermore, the lung metastases in HPSE-miRNA2 group were grade I (≤20 cells) or grade II (20–50 cells), while those in the negative control group or mock group were grade III (50–100 cells) or grade IV (>100 cells) (Figure 4C).

### Demonstration of the Specificity of HPSE-miRNA2

**Figure 5:**
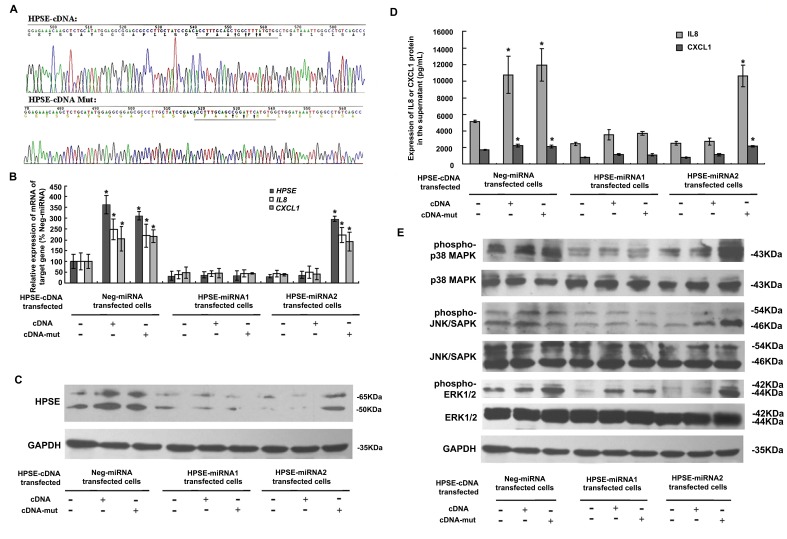
Restoration of HPSE functionality by HPSE RNAi rescue. (A) The sequencing results of the wild type and multisite mutant HPSE cDNA. Three nucleotide substitutions were introduced into the HPSE-miRNA2 hybridizing sequence (5′-CCTTTGCAGCTGGCTTTATGT-3′), which was verified by sequencing. (B-D) The levels of both *HPSE* mRNA and protein were restored in cells of the HPSE-miRNA2 group transfected with mutant HPSE cDNA, similar to those of Neg-miRNA cells transfected with the wild type or mutant HPSE cDNA. Furthermore, mutant cDNA transfection for miRNA rescue also increased *IL8* and *CXCL1* mRNA and protein levels. However, in HPSE-miRNA1 stably transfected cells, neither the wild type HPSE cDNA nor the mutant HPSE cDNA could restore the expressions of *HPSE*, *IL8* and *CXCL1*. (*P<0.05 compared with the respective non-cotransfected cells). (E) Restoration of the HPSE-induced phosphorylation of MAPKs in the cells of the HPSE-miRNA2 group by mutant HPSE cDNA. Phosphorylation of MAPK p38 (first and second panel), JNK/SAPK (third and fourth panel), and ERK1/2 (fifth and sixth panel) was monitored by western blotting.

RNA interference rescue was used [22] to normalize artificial miRNA-induced depletion of HPSE. We used multisite-directed mutagenesis and introduced three nucleotide substitutions within the HPSE-miRNA2 hybridizing sequence, while retaining the amino acid identity of the wild-type protein (Figure 5A). As shown in Figure 5B and C, introduction of the mutated HPSE cDNA in HPSE-miRNA2 stably transfected cells not only rescued the miRNA-induced HPSE inhibition but also increased *HPSE* mRNA levels (*P*<0.0001), similar to those of Neg-miRNA cells transfected with wild type or mutant HPSE cDNA (*P*>0.05). Furthermore, the transfection of cDNA-mut for miRNA rescue also increased *IL8* and *CXCL1* mRNA levels (*P*<0.01), indicating HPSE could regulate *IL8* and *CXCL1* at the level of transcription, or prior to translation. With respect to a regulatory mechanism, HPSE-induced phosphorylation of MAPKs was also restored in cells of the HPSE-miRNA2 group transfected with mutant HPSE cDNA, but not with the wild type HPSE cDNA (Figure 5E). However, in the HPSE-miRNA1 stably transfected cells, neither the original HPSE cDNA nor the mutant HPSE cDNA could restore the expression of HPSE, and subsequently, to rescue the levels IL8 or CXCL1, and phosphorylation of MAPKs (Figure 5B–E).

## Discussion

Tumor invasion and metastasis is a multistep process that promotes the spread of the cancer from primary sites to distant locations and requires a critical event for the ability of tumor cells to degrade and penetrate the ECM and BM [23]. HPSE is an endoglycosidase involved in HPSGs cleavage, a key component of the ECM, BM, and cell surface proteoglycans, leading to ECM remodeling, which may facilitate the cellular invasiveness associated with cancer metastasis [24]–[28]. During the past decade, many clinical data have revealed that the overexpression of HPSE correlates with reduced postoperative survival and poorer prognosis of colorectal, pancreatic, bladder, gastric, and cervical cancer patients [24]–[28]. Similarly, HPSE up-regulation also correlates with increased lymph node and distant metastasis [24], [27], [29] and with microvessel density [24], [29], [30], providing strong clinical support for the prometastatic and proangiogenic features of HPSE and positioning HPSE as a potentially new and promising drug target.

Due to potential nonspecific activity and significant toxicity of HPSE inhibitors, RNAi targeting HPSE (HPSE-siRNA or HPSE-shRNA) has been developed. This HPSE RNAi leads to slow growth, reduced clonogenic capacity, and invasive potential of aggressive tumor cell lines [31], [32]. Recent reports demonstrated that embedding a shRNA in the context of a naturally occurring Pol II-driven miRNA transcript increases the flexibility of RNAi allowing for conditional and cell type-specific expression [17], [18]. Additionally, such miRNA-based RNAi systems displayed very efficient knockdown of gene expression, even at a single copy [13], [14]. Furthermore, such an approach could alleviate the toxicity resulting from interference with the endogenous miRNA pathway or induction of the interferon response [33], [34]. In the present study, we utilized the BLOCK-iT™ Pol II miR RNAi expression vectors containing the human cytomegalovirus immediate early promoter to allow high-level, constitutive miRNA expression in mammalian cells. The engineered pre-miRNA sequence structure is based on the murine miR-155 sequence [35], which is one of the most characterized and commonly used pre-miRNA backbones [36]. We designed three different sequences targeting the HPSE gene, and the artificial HPSE miRNA was cloned and expressed. The silencing effect of the artificial miRNA, verified by real-time PCR and western blotting, showed that both the HPSE protein and mRNA were down-regulated in HPSE miRNA transfected A375 cells, especially in the HPSE-miRNA1 and HPSE-miRNA2 transfected groups (Figure 1D and E), which indicated that the artificial miRNAs could reduce the cellular concentration of their target mRNA and protein level [37], [38]. It was interesting that in our miRNA rescue experiments, only the mutated HPSE cDNA could completely restore the HPSE expression in HPSE-miRNA2 transfected cells at the transcriptional and translational levels (Figure 5B and C), consistent with the results from the Qin LX group [20]. The Qin LX group also constructed artificial miRNAs against osteopontin (OPN) and selected the OPNi-3 as the most effective miRNA. However, OPNi-3M, a control pre-miRNA with two mismatch mutations, did not display translational repression on OPN. The results from our group as well as other groups have indicated that artificial miRNAs may commonly encode a perfectly complementary guide strand that causes gene inhibition via mRNA cleavage [20], [36], unlike endogenous miRNAs with complete or partial complementarity to the target gene. miR-1258, an endogenous miRNA, was recently reported to suppress breast cancer brain metastasis by targeting HPSE. Decreased cell invasion and numbers of brain metastasis by treatment with miR-1258 were just partly reversed by employing an expression vector containing human HPSE [39]. To our knowledge, the data described here represents the first successful application of artificial miRNA-mediated gene silencing to effectively reduce the levels of HPSE.

The accumulated evidence has revealed that HSPGs inhibit cellular invasion by promoting tight cell-cell and cell-ECM interactions and by maintaining the structural integrity and self-assembly of the ECM [40], [41]. Therefore, the excessive expression of HPSE therefore may accelerate tumor cell dissemination, enabling the penetration of cells through the ECM barrier and improving tumor cell adhesion to endothelial cells and the subendothelial ECM by cleaving HSPGs [42], [43]. In view of the possible influence of cellular proliferation and viability on the migration or invasion of cells, we selected 24 hours as the observation point; at this time point, the cells number did not differ significantly amongst the groups, even though cellular viability between HPSE miRNA groups and control groups differed. Expectedly, in the present study, HPSE miRNA was able to abolish the invasive properties of melanoma cells *in vitro*, including adhesion, migration, and invasion (Figure 2E–H), which was consistent with previous studies [31], [32]. Xenograft models further confirmed that the down-regulation of HPSE by HPSE miRNA led to an obvious inhibition of *in vivo* lung metastasis of A375 cells (Figure 4C and D). The inhibitory effect of HPSE miRNA on the metastasis of melanoma *in vitro* and *in vivo* may be partly correlated with its suppression on cellular viability and proliferation, as well as the known important role of HPSE on the metastasis of melanoma.

To identify whether the silencing of unpredicted genes was due to off-target effects of RNAi or due to a secondary effect of the knock-down of the target gene, global gene expression patterns were analyzed [44], [45]. Our gene microarray results showed that 205 overlapping genes were up- or down-regulated by both HPSE miRNAs that targeted different sequences of the *HPSE* mRNA. It was worth noting that chemokines of *IL8*, *CXCL1*, *CCL2*, and *CCL11* were found to be down-regulated in both HPSE miRNA groups, the former two chemokine levels were confirmed by real-time PCR and ELISA assay (Figure 3C). Chemokines are secreted, low-molecular-weight chemotactic proteins that regulate the trafficking of leukocytes, including neutrophils, macrophages, and lymphocytes, to inflammatory sites. The metastasis of tumor cells has also been revealed to adopt a chemokine-mediated homing mechanism, similar to that of leukocyte trafficking. Previous studies reported that melanoma cells express high levels of IL8 and CXCL1 and their receptors CXCR1/2 and that IL8/CXCL1 signaling directly promotes cell migration of tumor cells, which may be relevant to tumor invasion and metastasis [46], [47]. Furthermore, melanoma cells produced and secreted high levels of IL8, which attracts neutrophils and increases β-2 integrin expression on their surface, which then interacts with intercellular adhesion molecule-1 on melanoma cells to promote anchoring to the vascular endothelium [48].

To date, the mechanism by which HPSE facilitates the expression of chemokines is thought to involve the release of ECM-resident chemokines [50]. HPSG serves as a storage depot for various members of the heparin-binding family of growth factors, cytokines, and chemokines [2], [49], and the cleavage of HPGS by HPSE ultimately releases these proteins and converts them into bioactive mediators. However, the mechanism by which *IL8* or *CXCL1* gene expression is regulated by HPSE at the transcriptional level remains unknown. The MAPK pathway is constitutively activated in most melanomas and plays a major role in mediating the survival and progression of melanoma [50], [51]. In addition, p38 MAPK [52], JNK [53], and ERK [54] are involved in the regulation of *IL8* expression in a variety of cell types. HPSE could promote phosphorylation of signaling molecules such as Akt and Src, facilitating gene transcription and phosphorylation of selected Src substrates [43], [55], whereas HPSE silencing was accompanied by reduced EGFR and Src phosphorylation levels [56]. Similarly, p38 MAPK activation may also be mediated by HPSE, resulting in the enhanced transcription of genes such as vascular endothelial growth factor [51], tissue factor (TF) [57], and cyclooxygenase-2 [58]. Herein, we provided evidence that the knockdown of HPSE with a HPSE miRNA reduced *IL8* and *CXCL1* in melanoma cells at both the transcriptional and translational levels (Figure 3C). In addition to release by HPSE, the gene expression of *IL8* and *CXCL1* may be mediated by the HPSE-induced phosphorylation of the p38 MAPK, JNK, and ERK pathway (Figure 3D). In RNAi rescue experiments to corroborate the specificity of the HPSE miRNA, the expression of *IL8* and *CXCL1* and the phosphorylation of MAPK were upregulated concordantly when the miRNA knockdown was rescued by an incompatible, mutated miRNA HPSE cDNA (Figure 5D and E). Thus, we inferred that the HPSE miRNA could block the expression of *IL8* and *CXCL1*, thus impairing the effect of *IL8* and *CXCL1* on the migration and invasion of tumor cells (Figure S5).

In conclusion, our data suggests that artificial miRNA driven by the Pol II cytomegalovirus promoter may inhibit the expression of the HPSE protein and mRNA effectively, resulting in decreased invasion properties of melanoma cells *in vitro* and *in vivo*. Moreover, inhibition of HPSE by miRNA was able to down-regulate *IL8* and *CXCL1* not only at the translational level, but also at the transcriptional level by the attenuation of MAPK phosphorylation, indicating that miRNAs could be useful tools to study gene function. However, there are still many problems that need to be addressed, including i) whether shRNAs targeting HPSE, based on an miRNA scaffold, are more efficient and safer compared with HPSE-shRNAs, siRNAs or inhibitors, ii) whether such HPSE miRNAs can be expressed in a conditional or tissue-specific manner *in vitro* or *in vivo* by the introduction of tissue-specific promoters, iii) whether there are suitable systems, such as optimized viral vectors or modified liposomes, to deliver the artificial miRNAs *in vivo*.

## Supporting Information

Figure S1 **The process of multisite mutagenesis of HPSE cDNA.** The following reactions were set up to produce mutated HPSE mRNA that is not degraded by HPSE-miRNA2. (A) Three nucleotide substitutions were introduced into the HPSE-miRNA2 hybridizing sequence (5′-CCTTTGCAGCTGGCTTTATGT-3′), which retained the amino acid identity of the wild-type protein. (B-C) The PCR reaction system, as shown in B, was set up to produce mutated HPSE cDNA, under the PCR condition shown in C. (D) The PCR mutagenesis reaction products were digested with 1.0 µL Dpn I at 37°C for 4 hours and were subsequently used for the transformation of TOP10 bacteria transformation. (E) DNA from positive colonies was isolated using a purification kit, digested by EcoRI and XhoI restriction enzymes and verified for correctness by electrophoresis, and sequenced (shown in Figure 5A). These mutated HPSE cDNAs were used for co-transfecting Neg-miRNA, HPSE-miRNA1 and HPSE-miRNA2 transfected cells for RNAi rescue.
(TIF)

Figure S2 **The correctness of HPSE-miRNA1, HPSE-miRNA2 and HPSE-miRNA3 were confirmed by sequencing.**
(TIF)

Figure S3 **HPSE miRNA down-regulated the expression of HPSE and inhibited adhesion, migration and invasion of HeLa cells.** (A) Quantitative real-time PCR results showed that the expression of *HPSE* mRNA in HeLa cells transfected with HPSE-miRNA1 was down-regulated compared to the parental cells and the Neg-miRNA transfected cells. (B) Representative blots were shown from three independent experiments with identical results. The expression of the HPSE protein of HeLa cells transfected with HPSE-miRNA1 was down-regulated compared to the parental cells and the Neg-miRNA group. (C) Cell-Matrigel adhesion assay. The adhesive ability of HeLa cells transfected with HPSE-miRNA1 was obviously inhibited compared to the parental cells and the Neg-miRNA group. (D) Representative images of invasive cells with HPSE-miRNA1, Neg-miRNA and parental cells from the Matrigel invasion assay (H&E staining, magnification of 40×10). (E) Representatives of migration cells from different groups in wound healing assays. The parental HeLa cells, and cells transfected with the Neg-miRNA or the HPSE-miRNA1, were seeded in 24-well plates at 2.5×10^5^ per well in a growth medium to form a confluent monolayer. Then a single scratch wound was created using a micropipette tip and cells were washed with phosphate-buffered saline to remove cell debris, supplemented with assay medium without serum. The images were captured with a microscope using a 10 × objective at 0 and 48 hours post-wounding. (F) Diagram of migrative cells or invasive cells as determined by the would healing assay or the Matrigel invasion assay. The number of migrative and invasive number of HeLa cells transfected with HPSE-miRNA1 was much less than that of either control group. (^†^ *P*<0.05 compared with the parental cells; *P<0.05 compared with the Neg-miRNA transfected cells).
(TIF)

Figure S4 **Differential gene expression caused by the knockdown of HPSE and its related functional pathway.** (A) Differential gene expression between the Neg-miRNA and the HPSE-miRNA1 or HPSE-miRNA2 transfected A375 cells by gene microarray analysis using Phalanx Human OneArray™ slides. (B) 205 overlapping genes were analyzed for functional annotation by DAVID.
(TIF)

Figure S5 **Schematic representation of the hypothetical molecular mechanisms, by which the HPSE miRNA regulates the expression of** ***IL8*** **and** ***CXCL1*** **and participates in the inhibition of melanoma migration and invasion.** (A) Schematic diagram of the HPSE-induced IL8/CXCL1/MAPK pathway. (B) The HPSE miRNA blocked the expression of *IL8* and *CXCL1* and participated in the inhibition of melanoma migration and invasion. (C) Schematic drawing of distant metastasis of melanoma induced by IL8 or CXCL1.
(TIF)
