# Supplementary figures and images for: An Artificial miRNA against HPSE Suppresses Melanoma Invasion Properties, Correlating with a Down-Regulation of Chemokines and MAPK Phosphorylation
Source: PLoS One. 2012 Jun 15;7(6):e38659. doi: 10.1371/journal.pone.0038659 (PMC3376136; doi:10.1371/journal.pone.0038659)

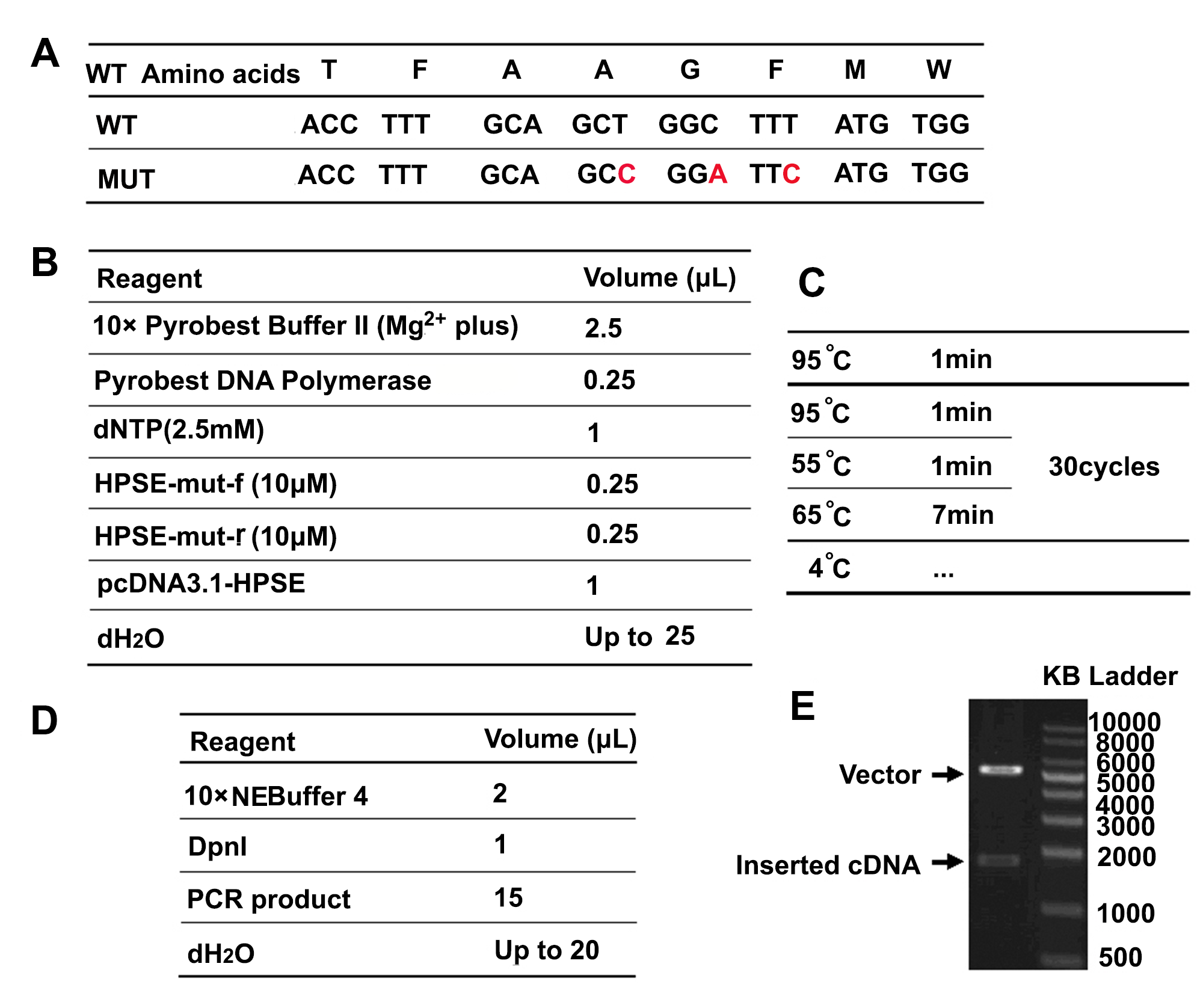

Supplement: Figure S1 — The process of multisite mutagenesis of HPSE cDNA. The following reactions were set up to produce mutated HPSE mRNA that is not degraded by HPSE-miRNA2. (A) Three nucleotide substitutions were introduced into the HPSE-miRNA2 hybridizing sequence (5′-CCTTTGCAGCTGGCTTTATGT-3′), which retained the amino acid identity of the wild-type protein. (B-C) The PCR reaction system, as shown in B, was set up to produce mutated HPSE cDNA, under the PCR condition shown in C. (D) The PCR mutagenesis reaction products were digested with 1.0 µL Dpn I at 37°C for 4 hours and were subsequently used for the transformation of TOP10 bacteria transformation. (E) DNA from positive colonies was isolated using a purification kit, digested by EcoRI and XhoI restriction enzymes and verified for correctness by electrophoresis, and sequenced (shown in Figure 5A). These mutated HPSE cDNAs were used for co-transfecting Neg-miRNA, HPSE-miRNA1 and HPSE-miRNA2 transfected cells for RNAi rescue. (TIF) [file pone.0038659.s001.tif]

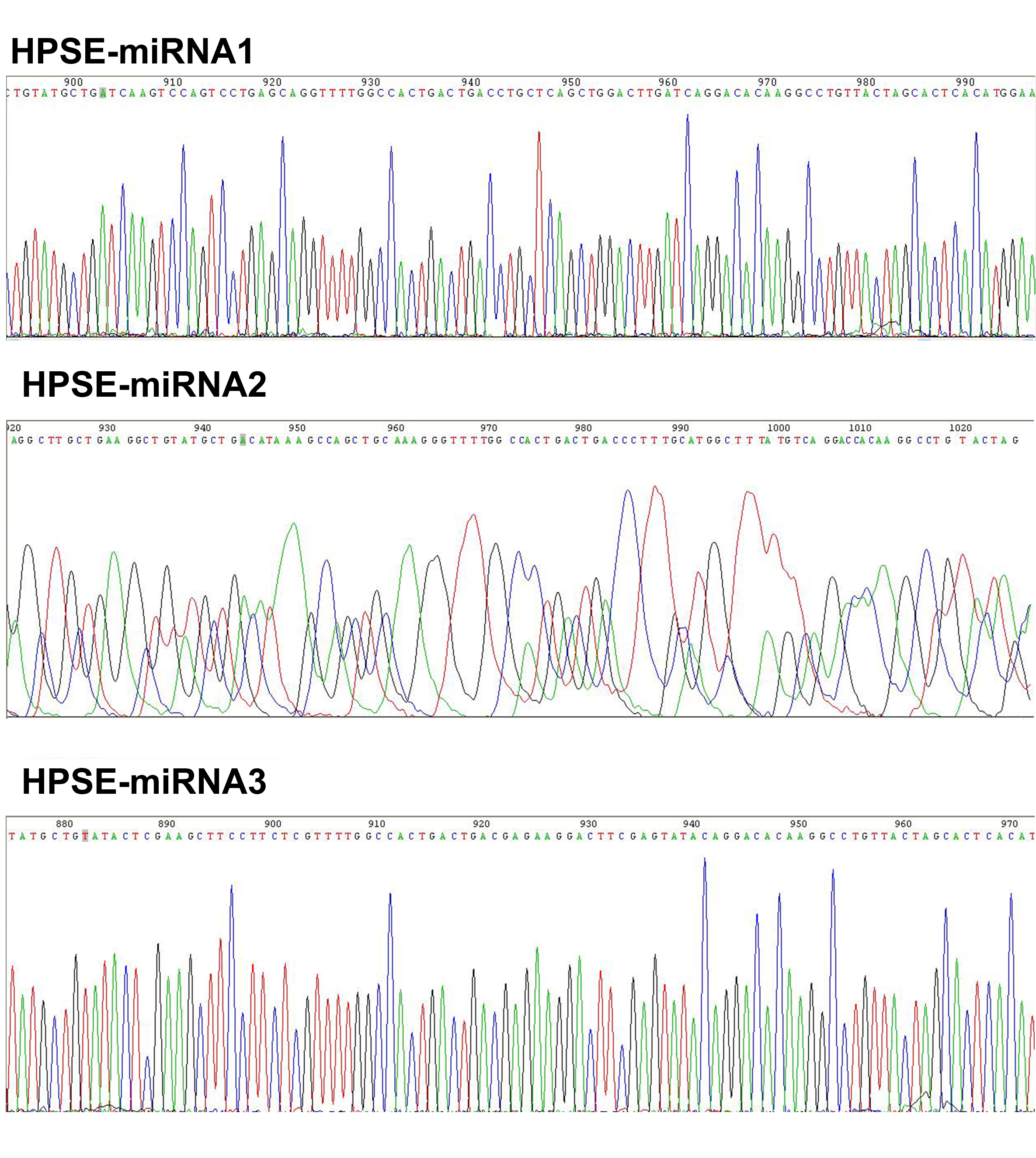

Supplement: Figure S2 — The correctness of HPSE-miRNA1, HPSE-miRNA2 and HPSE-miRNA3 were confirmed by sequencing. (TIF) [file pone.0038659.s002.tif]

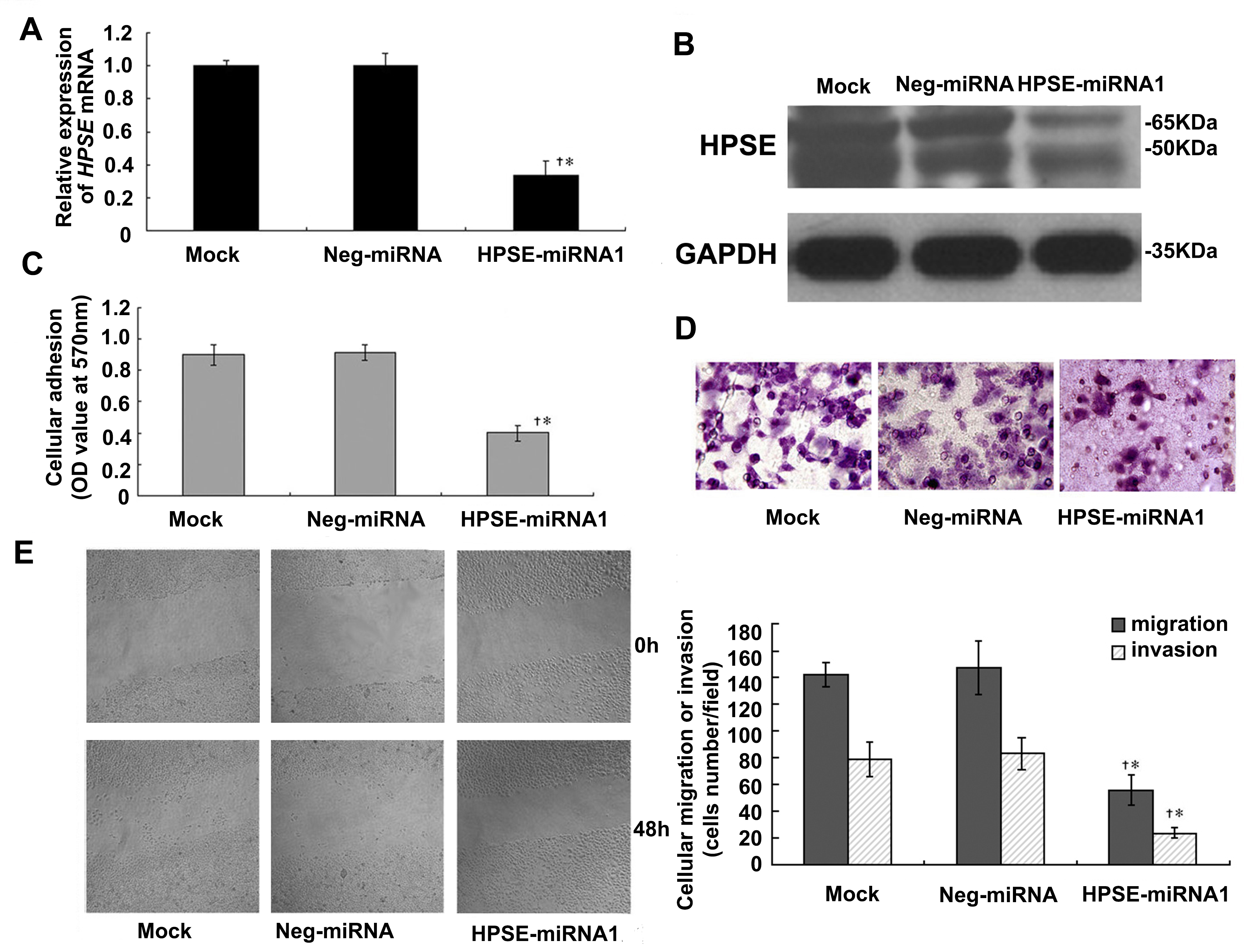

Supplement: Figure S3 — HPSE miRNA down-regulated the expression of HPSE and inhibited adhesion, migration and invasion of HeLa cells. (A) Quantitative real-time PCR results showed that the expression of HPSE mRNA in HeLa cells transfected with HPSE-miRNA1 was down-regulated compared to the parental cells and the Neg-miRNA transfected cells. (B) Representative blots were shown from three independent experiments with identical results. The expression of the HPSE protein of HeLa cells transfected with HPSE-miRNA1 was down-regulated compared to the parental cells and the Neg-miRNA group. (C) Cell-Matrigel adhesion assay. The adhesive ability of HeLa cells transfected with HPSE-miRNA1 was obviously inhibited compared to the parental cells and the Neg-miRNA group. (D) Representative images of invasive cells with HPSE-miRNA1, Neg-miRNA and parental cells from the Matrigel invasion assay (H&E staining, magnification of 40×10). (E) Representatives of migration cells from different groups in wound healing assays. The parental HeLa cells, and cells transfected with the Neg-miRNA or the HPSE-miRNA1, were seeded in 24-well plates at 2.5×10^5^ per well in a growth medium to form a confluent monolayer. Then a single scratch wound was created using a micropipette tip and cells were washed with phosphate-buffered saline to remove cell debris, supplemented with assay medium without serum. The images were captured with a microscope using a 10 × objective at 0 and 48 hours post-wounding. (F) Diagram of migrative cells or invasive cells as determined by the would healing assay or the Matrigel invasion assay. The number of migrative and invasive number of HeLa cells transfected with HPSE-miRNA1 was much less than that of either control group. († P<0.05 compared with the parental cells; *P<0.05 compared with the Neg-miRNA transfected cells). (TIF) [file pone.0038659.s003.tif]

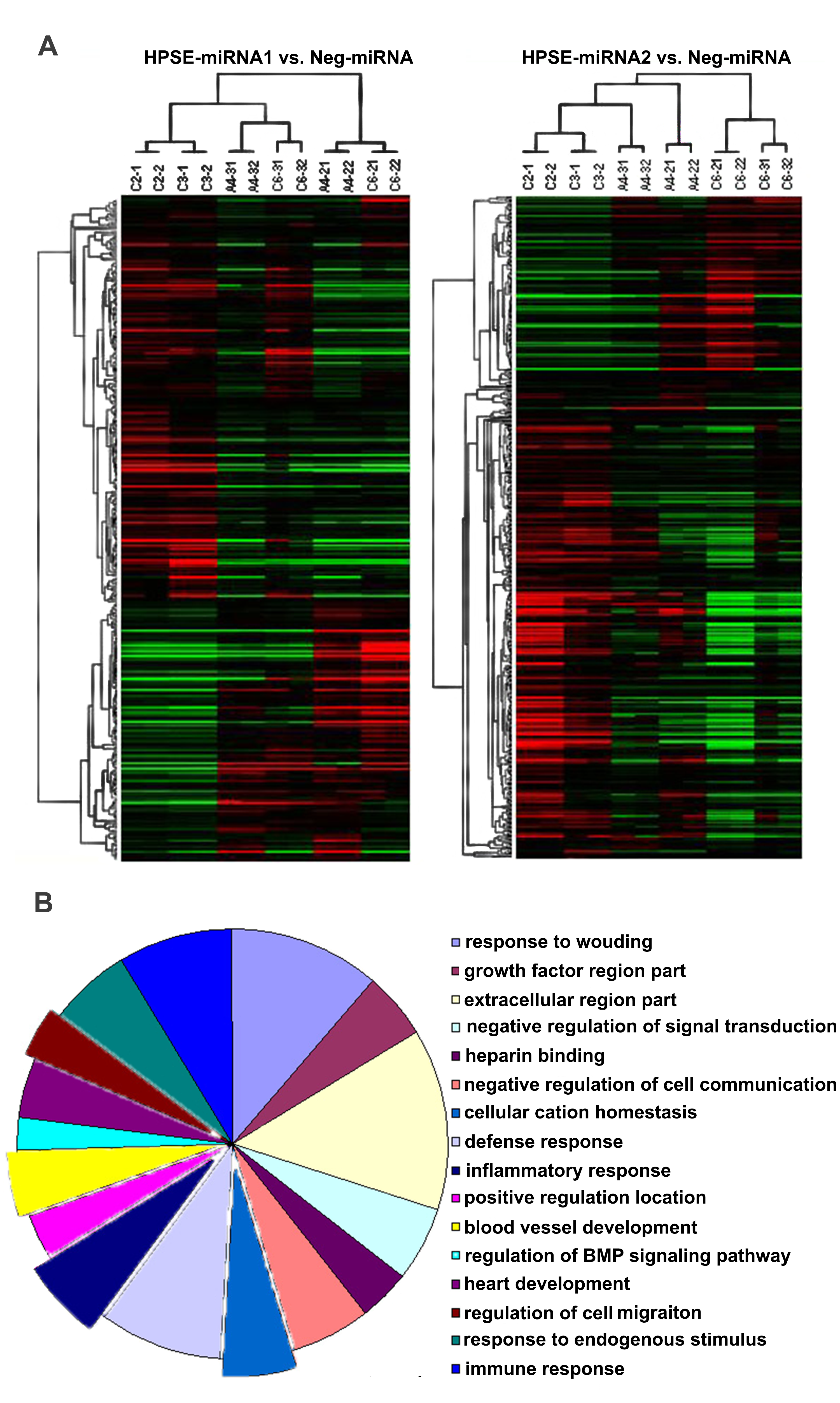

Supplement: Figure S4 — Differential gene expression caused by the knockdown of HPSE and its related functional pathway. (A) Differential gene expression between the Neg-miRNA and the HPSE-miRNA1 or HPSE-miRNA2 transfected A375 cells by gene microarray analysis using Phalanx Human OneArray™ slides. (B) 205 overlapping genes were analyzed for functional annotation by DAVID. (TIF) [file pone.0038659.s004.tif]

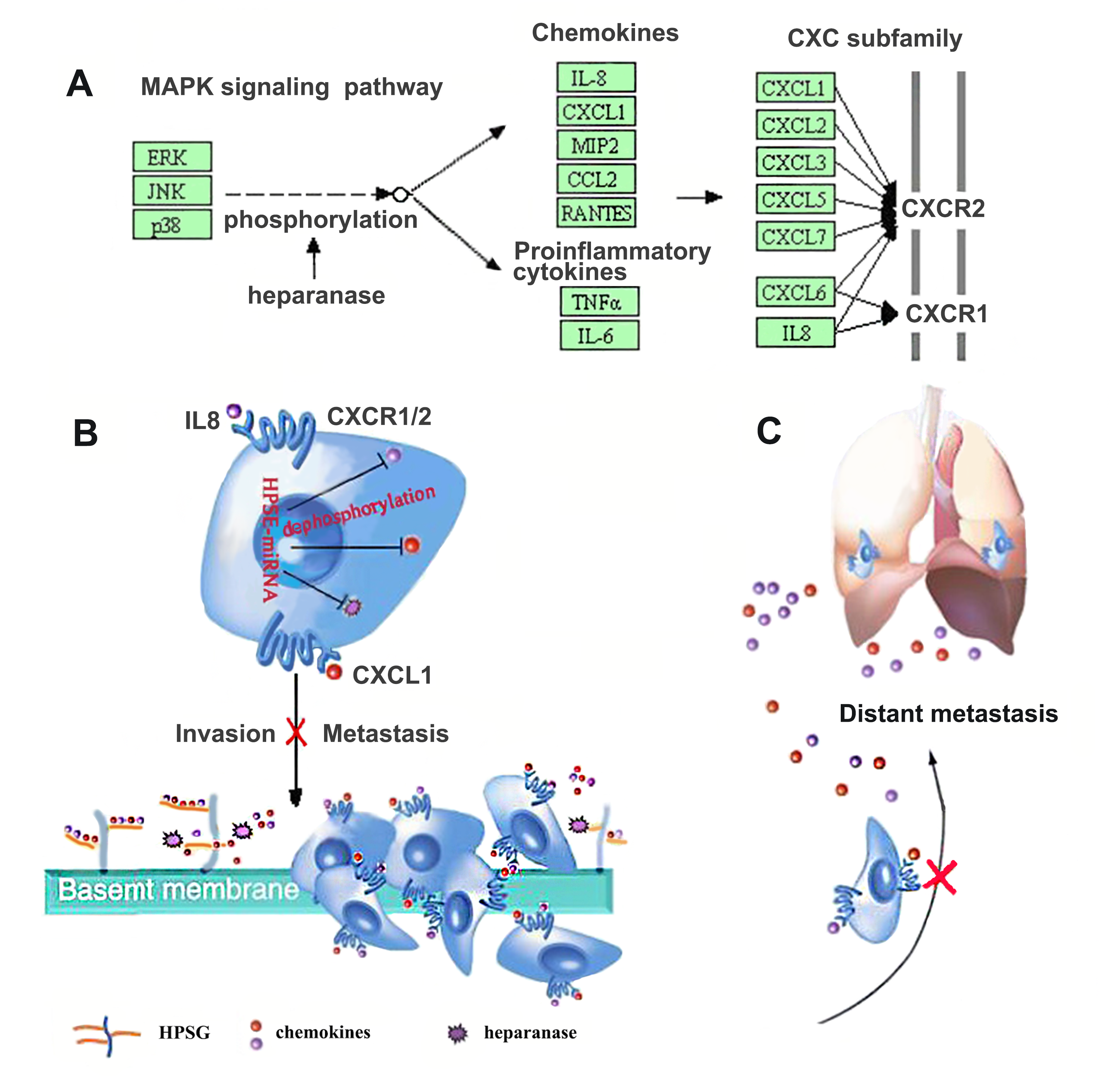

Supplement: Figure S5 — Schematic representation of the hypothetical molecular mechanisms, by which the HPSE miRNA regulates the expression of IL8 and CXCL1 and participates in the inhibition of melanoma migration and invasion. (A) Schematic diagram of the HPSE-induced IL8/CXCL1/MAPK pathway. (B) The HPSE miRNA blocked the expression of IL8 and CXCL1 and participated in the inhibition of melanoma migration and invasion. (C) Schematic drawing of distant metastasis of melanoma induced by IL8 or CXCL1. (TIF) [file pone.0038659.s005.tif]
